# Supplementary figures and images for: Ectopic Expression of PsnNAC090 Enhances Salt and Osmotic Tolerance in Transgenic Tobacco
Source: Int J Mol Sci. 2023 May 19;24(10):8985. doi: 10.3390/ijms24108985 (PMC10218818; doi:10.3390/ijms24108985)

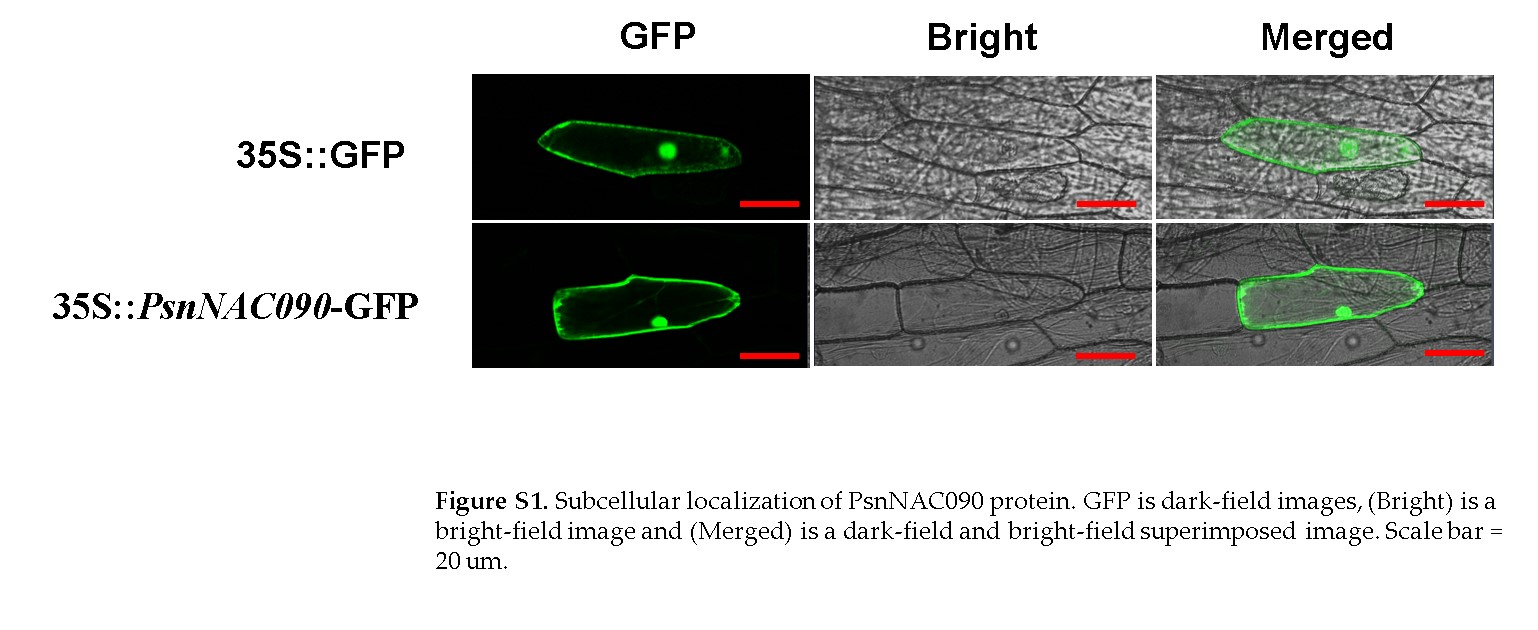

Supplement: Supplementary file 1 [file ijms-24-08985-s001.zip › FigS1.jpg]

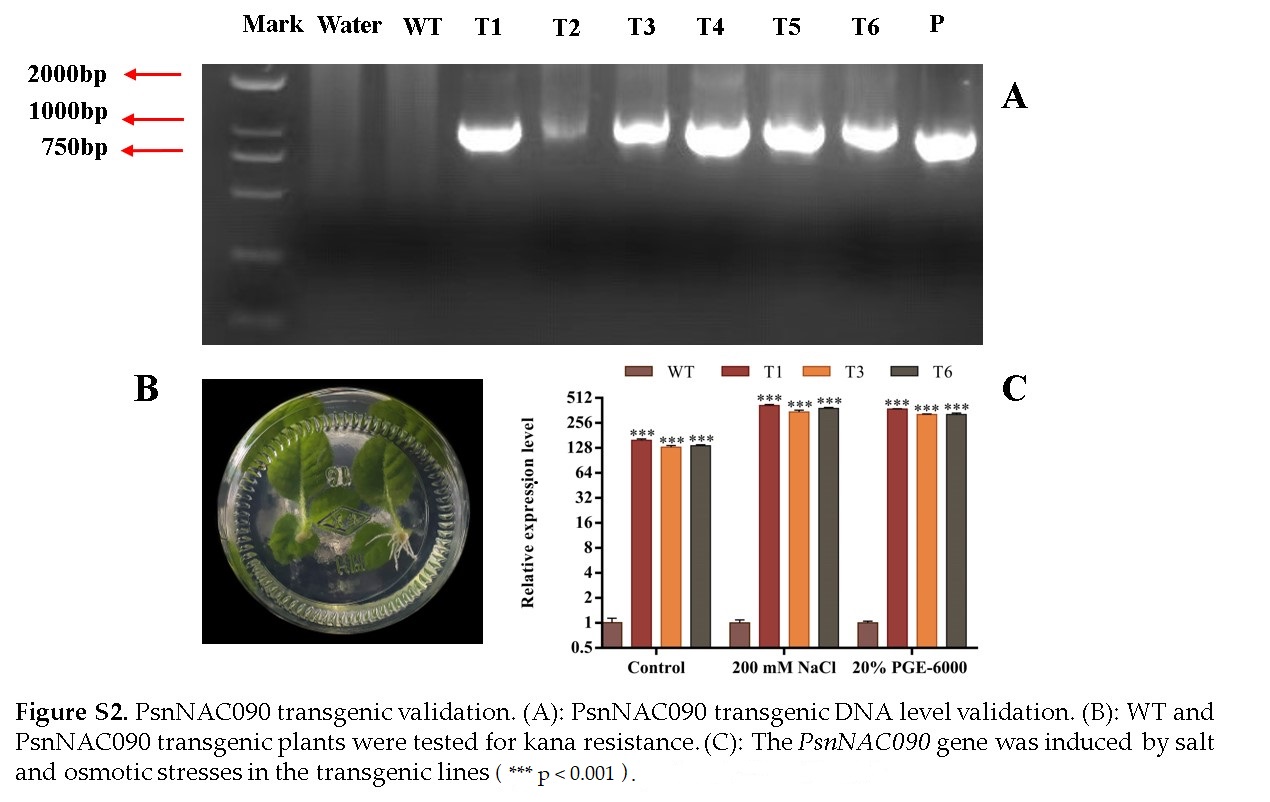

Supplement: Supplementary file 1 [file ijms-24-08985-s001.zip › FigS2.jpg]
